# Pyrintegrin Induces Soft Tissue Formation by Transplanted or Endogenous Cells

**DOI:** 10.1038/srep36402

**Published:** 2017-01-27

**Authors:** Bhranti S. Shah, Mo Chen, Takahiro Suzuki, Mildred Embree, Kimi Kong, Chang H. Lee, Ling He, Lusai Xiang, Jeffrey A. Ahn, Sheng Ding, Jeremy J. Mao

**Affiliations:** 1 Center for Craniofacial Regeneration, Columbia University Medical Center, 630 W. 168 St.-PH7E, New York, NY 10032, USA; 2 Department of Biomedical Engineering, Columbia University, 500 W. 120th St., Mudd 510, New York, NY 10027, USA; 3 Guanghua School of Stomatology, Hospital of Stomatology, Sun Yat-sen University, 56 Lingyuanxi Road, Guangzhou 510055, China; 4 Gladstone Institute of Cardiovascular Disease, Department of Pharmaceutical Chemistry, University of California San Francisco, 505 Parnassus Ave, San Francisco, CA 94122, USA

## Abstract

Focal adipose deficiency, such as lipoatrophy, lumpectomy or facial trauma, is a formidable challenge in reconstructive medicine, and yet scarcely investigated in experimental studies. Here, we report that Pyrintegrin (Ptn), a 2,4-disubstituted pyrimidine known to promote embryonic stem cells survival, is robustly adipogenic and induces postnatal adipose tissue formation *in vivo* of transplanted adipose stem/progenitor cells (ASCs) and recruited endogenous cells. *In vitro*, Ptn stimulated human adipose tissue derived ASCs to differentiate into lipid-laden adipocytes by upregulating peroxisome proliferator-activated receptor (PPARγ) and CCAAT/enhancer-binding protein-α (C/EBPα), with differentiated cells increasingly secreting adiponectin, leptin, glycerol and total triglycerides. Ptn-primed human ASCs seeded in 3D-bioprinted biomaterial scaffolds yielded newly formed adipose tissue that expressed human PPARγ, when transplanted into the dorsum of athymic mice. Remarkably, Ptn-adsorbed 3D scaffolds implanted in the inguinal fat pad had enhanced adipose tissue formation, suggesting Ptn’s ability to induce *in situ* adipogenesis of endogenous cells. Ptn promoted adipogenesis by upregulating PPARγ and C/EBPα not only in adipogenesis induction medium, but also in chemically defined medium specifically for osteogenesis, and concurrently attenuated Runx2 and Osx via BMP-mediated SMAD1/5 phosphorylation. These findings suggest Ptn’s novel role as an adipogenesis inducer with a therapeutic potential in soft tissue reconstruction and augmentation.

Focal soft tissue defects frequently represent substantial loss of adipose tissue such as that resulting from breast cancer resection, facial trauma or congenital anomalies^1^,^2^,^3^. Despite a significant challenge for surgical reconstruction, focal adipose deficiency is scarcely investigated in experimental approaches of tissue regeneration^4^,^5^. Adipose tissue regeneration offers several advantages over the current treatment modalities such as autologous grafting and commercially available fillers such as fibrin, hyaluronic acid, collagen for treating focal tissue defects^4^,^6^. Several stem cell sources have been utilized for soft tissue engineering: bone marrow^7^,^8^,^9^,^10^,^11^, abdominal fat^8^,^12^,^13^,^14^,^15^,^16^,^17^, ligament etc.^15^. Progenitor cells from adipose tissue consistently differentiate into lipid-laden adipocytes *in vitro*^2^,^8^,^10^,^11^,^16^.

Adipogenic differentiation of stem/progenitor cells is achieved using adipogenic induction medium, typically with a combination of insulin, dexamethasone, indomethacin and methylisobutylxanthine (IBMX), which elevates intracellular cAMP levels, in the presence of fetal bovine serum^2^,^10^,^11^,^16^,^17^,^18^. Adipogenic differentiation of stem/progenitor cells is primarily gated by two families of transcription factors, peroxisome proliferator-activated receptor-γ (PPARγ) and CCAT/enhancer binding protein (CEBPs)^19^,^20^,^21^. PPARγ, a member of the nuclear hormone receptor family, is expressed upon stem/progenitor cell commitment to differentiate towards adipocytes^22^,^23^. The C/EBPs belong to the basic-leucine zipper class of transcription factors^24^,^25^. C/EBPα, β, δ isoforms form a homo- and/or heterodimer configuration via a highly conserved bZIP domain^26^,^27^. C/EBPα alone or with PPARγ promotes adipose cell differentiation of fibroblasts or myoblasts^28^,^29^. Therefore, orchestrated regulation between PPARγ and C/EBPs may be crucial in initiating and maintaining the differentiated state of adipocytes^23^,^26^.

Small molecules that regulate adipocyte differentiation have been explored, including phenamil^30^, harmine^31^, SR6452, a synthetic REV-ERB ligand^32^ and known PPARγ agonists such as Troglitazone^33^,^34^, Rosiglitazone^35^,^36^,^37^, Pioglitazone^38^,^39^,^40^. The majority of these small molecules act via PPARγ pathway and facilitate adipogenesis *in vitro*. Activation of PPARγ receptor has been shown to promote adipogenesis and inhibit osteogenesis in bone marrow stem/stromal cells^41^. In this report, we discovered that Pyrintegrin (Ptn), a small molecule identified through high-throughput screening for its ability to promote the survival of human embryonic stem cells^42^, not only attenuated Runx2 and Osx via BMP-mediated SMAD1/5 phosphorylation, but also concurrently upregulates PPARγ and C/EBPα. *In vivo*, Ptn-treated human ASCs in 3D-bioprinted scaffolds, when transplanted in the dorsum of athymic mice, yielded ectopically formed adipose tissue that expressed human PPARγ. Remarkably, Ptn-adsorbed collagen gel implanted in the inguinal fat pad promoted adipogenesis formed by host endogenous cells, suggesting its ability to induce *in situ* adipogenesis without the need for cell transplantation.

## Results

### Pyrintegrin promotes adipogenic differentiation of human adipose stem/progenitor cells *in vitro*

**Figure 1:**
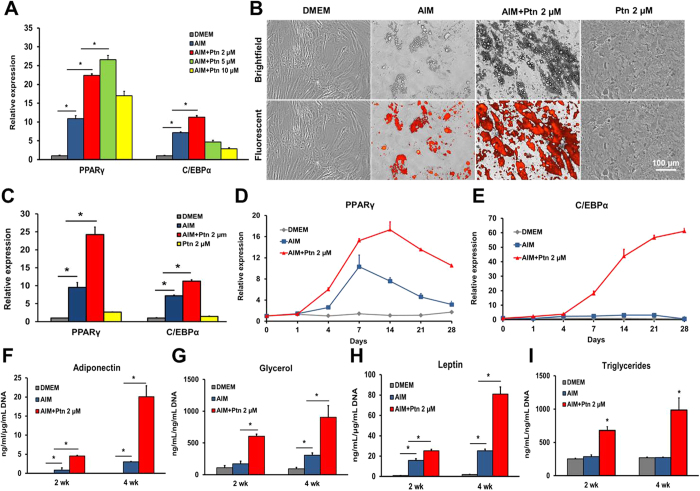
Pyrintegrin enhances adipogenesis of human adipose stem/progenitor cells *in vitro*. (**A**) Ptn dose optimization by measuring PPARγ and C/EBPα expression in DMEM, AIM, AIM containing varying doses of Ptn 2-, 5- and 10-μM in hASC cultures after 4 days. Human GAPDH was used as the housekeeping gene and values representing mRNA of the DMEM group were defined as 1. AIM: adipogenesis induction medium; Ptn: Pyrintegrin. (**B**) Lipid accumulation in DMEM, AIM, AIM+Ptn 2-μM and Ptn 2-μM alone hASC cultures over 4 wks. Scale bar: 100 μm. (**C**) Relative mRNA expression of PPARγ and C/EBPα in DMEM, AIM, AIM+Ptn 2-μM and Ptn 2-μM alone hASC cultures at day 7. (**D,E**) Time-course real time RT-PCR analysis of PPARγ and C/EBPα expression of DMEM, AIM and AIM+Ptn 2-μM treated hASC cultures over 28 days. (**F–I**) Supernatant analysis of adiponectin, glycerol, leptin and triglycerides measured from DMEM, AIM and AIM+Ptn 2-μM treated hASC cultures at 2 and 4 wks. Data are expressed as mean ± S.D. *P < 0.05.

Passage 4–5 ASCs were plated at a density of 5 × 10^3^ cells per cm^2^ and culture-expanded in 24-well plates till confluence, followed by adipogenic differentiation per our prior methods^20^,^21^,^28^. To optimize Ptn doses, human ASCs were cultured in DMEM, adipogenesis induction medium (AIM), and Ptn-supplemented AIM at 2-, 5- or 10-μM. PPARγ and C/EBPα expression peaked at 2-μM Ptn, significantly more than DMEM, AIM alone and 5 or 10 μM Ptn-supplemented AIM (Fig. 1A). Hence, 2-μM Ptn dose was adopted in subsequent experiments. Lipid droplets were stained with fluorescent lipid dye per manufacturer’s protocol following 4-wk exposure to adipogenesis induction medium (AIM). Contrary to a lack of adipogenesis in DMEM, lipid accumulation occurred in AIM (Fig. 1B). Remarkably, Ptn at 2- μM concentration robustly augmented lipid accumulation (Fig. 1B). However, Ptn alone failed to induce adipogenesis (Fig. 1B), suggesting that Ptn only augments adipogenic differentiation in the presence of adipogenic supplements in AIM. Quantitatively, Ptn upregulated PPARγ and C/EBPα in AIM, significantly more than AIM alone and DMEM (Fig. 1C). Again, Ptn alone failed to upregulate PPARγ or C/EBPα (Fig. 1C). Human ASCs isolated from different donors were tested similarly, and were shown to exhibit similar lipid accumulation differences (data not shown).

Upon exposure to 2 μM Ptn-supplemented AIM, PPARγ expression began to elevate by Day 4 and peaked between 7 and 14 days, followed by a decline at 21 days (Fig. 1D); C/EBPα expression began to increase by Day 4, and continued to escalate up to the tested 21 days (Fig. 1E), suggesting Ptn’s concerted and sequential effects on PPARγ and C/EBPα expression during adipogenic differentiation in ASCs. Quantitatively, ASCs secreted significantly more levels of adiponectin, leptin, total triglycerides and glycerol (Fig. 1F–I, respectively) upon 2- or 4-wk exposure to 2-μM Ptn-supplemented AIM than cells cultured in DMEM or AIM.

### Pyrintegrin primed human adipose stem/progenitor cells yielded adipose tissue formation *in vivo*

**Figure 2:**
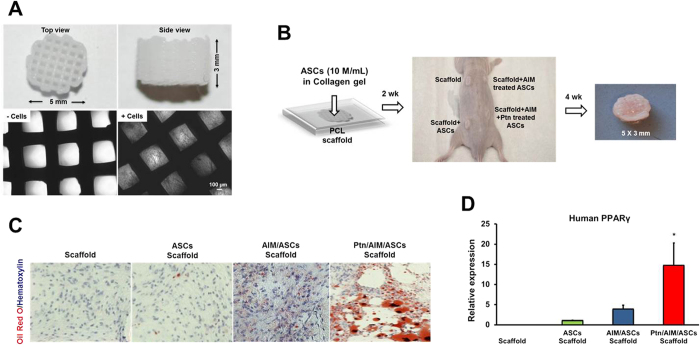
Pyrintegrin induces adipogenesis of transplanted human adipose stem/progenitor cells *in vivo*. (**A**) Polycaprolactone (PCL) particles were melted and 3D-printed as cylinders, with top and side views and microscopic images without and with cells seeded in microchannels. (**B**) Infusion of human adipose derived stem cells (hASCs) in collagen gel into PCL microchannels, followed by implantation in the dorsum of athymic mice and retrieved in 4 wks, with a representative sample shown. (**C**) Representative histology images of *in vivo* retrieved samples stained for lipids by Oil-Red-O dye and nucleus by hematoxylin stain. (**D**) q RT-PCR analysis of human PPARγ, of *in vivo* retrieved samples. Scale bar: 100 μm. Data are expressed as mean ± S.D. *P < 0.05.

A microporous scaffold was fabricated by 3D layer-by-layer apposition using 100 wt% poly-ε-caprolactone (PCL) with ~65 kDa molecular weight per our prior approach^43^,^44^. Cylindrical PCL scaffolds were fabricated with an overall dimension of 5 × 3 mm^2^ (diameter × height) (Fig. 2A) and with interconnecting microchannels as conduits to promote tissue regeneration. Interlaid strand diameters of 250–300 μm and interconnecting microchannel diameters of 400–500 μm were selected to facilitate host cell infiltration and angiogenesis per our previous data^43^,^44^ (Fig. 2A). Human ASCs were culture-expanded in DMEM, AIM, or 2-μM Pyrintegrin supplemented AIM for 2 wks. A total of 10 × 10^6^ cells/mL were homogenously seeded in 3 mg/mL type I collagen solution, and infused into the scaffold’s microchannels (Fig. 2B), followed by incubation at 37 °C for 1 h to allow gelation. Subcutaneous pockets were surgically created in 8–9-wk-old athymic mice (Fig. 2B). Cell-infused or cell-free PCL scaffolds were implanted subcutaneously in the dorsum. Following 4-wk implantation, tissue ingrowth was apparent in all groups (N = 6/group) as exemplified in Fig. 2B. Cell infiltration occurred in all groups: scaffold alone, ASC-infused scaffolds, AIM-primed ASC scaffolds and Ptn- and AIM-treated ASC scaffolds (Fig. 2C). A moderate amount of adipose tissue was found in Ptn- and AIM-treated ASCs as revealed by Oil-red O staining (Fig. 2C). Human PPARγ expression was most pronounced in Ptn- and AIM-treated ASC scaffolds, significantly more than AIM-primed ASC scaffolds, ASC-infused scaffolds or scaffold only groups (Fig. 2D).

### Pyrintegrin promotes adipogenesis from host endogenous cells without cell transplantation

**Figure 3:**
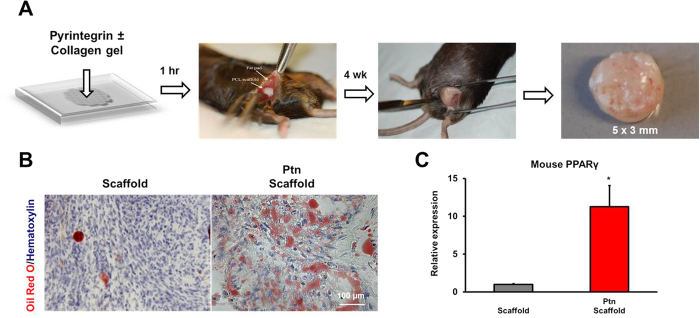
Pyrintegrin enhances endogenous adipose tissue formation *in vivo*. (**A**) Pyrintegrin (Ptn) (10 μg/mL) was adsorbed in collagen gel that was infused in 3D-printed polycaprolactone (PCL) scaffold, followed by 1-h incubation at 37 °C and implantation in the inguinal fat pad of C57BL/6 mice. Representative sample image following scaffold retrieval after 4 wks. (**B**) Representative Oil-Red-O images of *in vivo* retrieved scaffold and Ptn-infused scaffold samples. (**C**) qRT-PCR analysis showing increased mouse PPARγ gene expression in Ptn-infused scaffold group. Scale bar: 100 μm. Data are expressed as means ± SD. *P < 0.05.

Next, we performed another *in vivo* experiment to determine whether Ptn promotes endogenous adipogenesis without cell transplantation. A total of 10-μg/mL Ptn was homogenously seeded in 3 mg/mL neutralized type I collagen solution (20 μL). Ptn-adsorbed or Ptn-free collagen solution was infused into PCL scaffold’s microchannels (Fig. 3A), followed by gelation for 1 h at 37 °C. Ptn-adsorbed or Ptn-free PCL scaffolds were implanted in the inguinal fat pad *in vivo* in C57BL/6 mice (N = 6/group) (Fig. 3A). Following 4-wk *in vivo* implantation, little adipose tissue was formed in the representative Ptn-free scaffolds (Fig. 3B). Remarkably, Ptn-adsorbed collagen gel induced formation of adipose tissue that was positive to Oil Red O (Fig. 3B). Mouse PPARγ expression with RNA extracted from *in vivo* harvested tissue within the scaffold’s microchannels was significantly higher in Ptn-adsorbed collagen gel scaffolds than Ptn-free collagen gel scaffolds (Fig. 3C). Given that no cells were transplanted, Ptn promotes endogenous adipogenesis in the native adipose environment.

### Pyrintegrin attenuates SMAD-dependent BMP signaling

**Figure 4:**
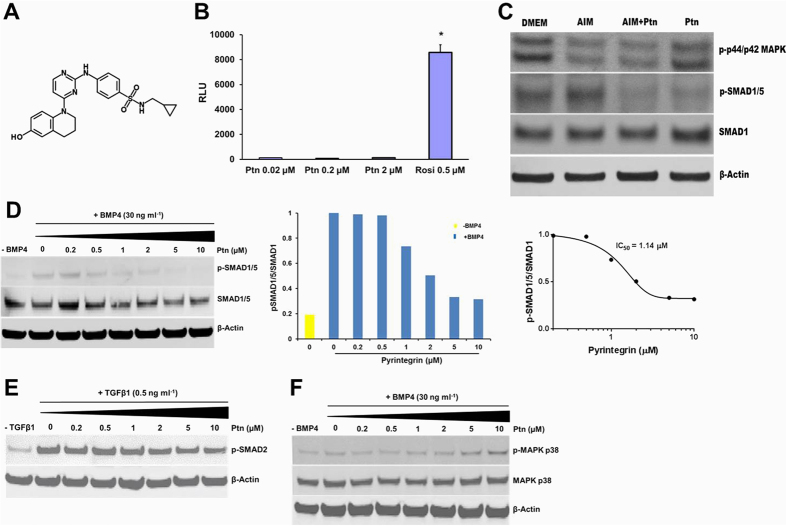
Pyrintegrin attenuates BMP-mediated SMAD activation. (**A**) Ptn chemical structure. (**B**) Dose response of human PPPARγ luciferase assay. RLU, relative light units. (**C**) MAPK p44/p42 and SMAD1/5 phosphorylation by immunoblot after pretreatment with DMEM, AIM, AIM + Ptn and Ptn alone for 1 h. Equivalent protein loading confirmed by total SMAD1 and β-actin. (**D**) Western blot and densitometry analysis of SMAD1/5 phosphorylation following Ptn pretreatment for 30 min followed by treatment with BMP4 for 30 min. Equivalent protein loading confirmed by detection of total SMAD1 and β-actin. Hill plot of the inhibition of BMP4-stimulated SMAD1/5/8 phosphorylation by incubating hASCs with Pyrintegrin. (**E**) SMAD2 phosphorylation treated with BMP4 showing virtually negative Ptn effect on BMP4-mediated SMAD2 activity. (**F**) Ptn attenuated BMP4-mediated MAPK p38 phosphorylation modestly at concentrations ≥ 5 μM. Data are expressed as means ± SD. *P < 0.05.

To explore Ptn signaling mechanisms, we first performed a human PPARγ luciferase receptor assay. PPARγ activity was unresponsive to Ptn stimulation at 0.02, 0.2 or 2 μM, but was promoted by Rosiglitazone, a known potent PPARγ agonists (Fig. 4A,B). Accordingly, we screened several known adipogenesis signaling pathways and found that Ptn down-regulated the phosphorylation of Smad1/5, a downstream BMP target (Fig. 4C). However, Ptn did not inhibit the phosphorylation of p42/44 MAPK (Fig. 4C), a downstream target of MEK1/2 pathway. To further explore whether Ptn acts through SMAD-dependent BMP signaling, hASCs were treated with no BMP4, or BMP4 + Ptn (0 to 10 μM) media. Both western blot and densitometry analysis confirm our finding that Ptn inhibited BMP4-mediated phosphorylation of BMP responsive SMAD1/5 in a dose-dependent manner (half maximal inhibitory concentration (IC_50_) = 1.14 μM), (Fig. 4D), but failed to attenuate SMAD2 activation by TGFβ1 at concentrations equal to or greater than those that were inhibitory to BMP signaling (Fig. 4E). Ptn activated phosphorylation of p38 MAPK at higher doses (5 and 10 μM), but not at lower concentrations (Fig. 4F) in the presence of BMP4. p38 MAPK is responsive to both cellular non-stress and stress stimuli signals^45^ and our results suggest that at higher doses of Ptn, the cells might be experiencing cytotoxic stress.

### Pyrintegrin attenuates osteogenesis and promotes adipogenesis

**Figure 5:**
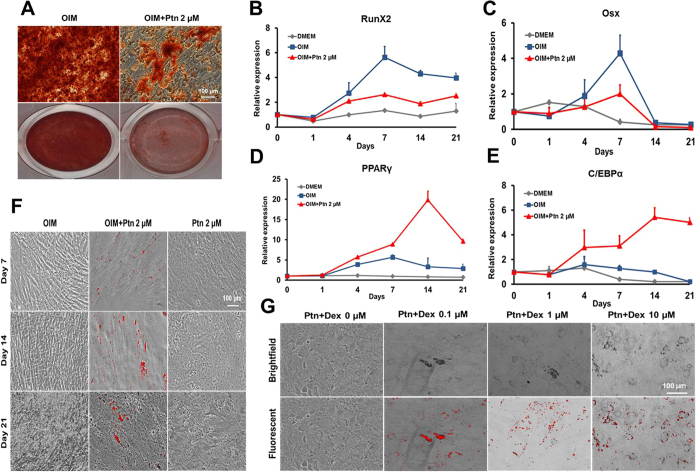
Pyrintegrin attenuates osteogenic differentiation *in vitro*. (**A**) Alizarin Red staining following treatment of adipose stem/progenitor cells (ASCs) in osteogenesis induction medium (OIM) and OIM+Pyrintegrin (Ptn) for 3 wks. (**B,C**) Time-course qPCR analysis of Runx2, Osx, PPARγ and C/EBPα mRNA expression measured over 21 days. (**F**) Lipid staining of hASCs treated with OIM, OIM+Ptn and Ptn alone at Days 7, 14 and 21. (**G**) Lipid staining of hASCs exposed to 2-μM Ptn and multiple dexamethasone doses (0, 0.1, 1 and 10 μM) for 21 days.

At 80–90% confluence, hASCs were treated with osteogenesis induction medium (OIM), and OIM containing 2-μM Ptn (Fig. 5A). In comparison with expected robust Alizarin Red staining with ASCs in OIM for 21 days, Ptn drastically attenuated Alizarin Red staining (Fig. 5A). By 21 days, Ptn significantly attenuated Runx2 and Osx (Fig. 5B,C). Ptn further upregulated PPARγ (Fig. 5D) and C/EBPα (Fig. 5E) even in chemically defined medium for osteogenesis. Ptn further stimulated lipid droplets in OIM at 7, 14 and 21 days (Fig. 5F). Importantly, Ptn alone failed to induce lipid accumulation (Fig. 5F). Given that Ptn induced adipogenesis in OIM but not in DMEM, we compared the known ingredients between osteogenesis induction medium and adipogenesis induction medium based on common protocols including the ones used in the present study, and found that the only common component is dexamethasone (Dex). With a postulation that Dex may have synergistic effect with Ptn in promoting adipogenesis, we found that indeed, 2-μM Ptn and Dex at 0.1-, 1- and 10-μM concentrations conjunctively promoted lipid accumulation for the tested 21 days (Fig. 5G), suggesting that Dex is likely the key ingredient in both osteogenesis and adipogenesis induction media that enables Ptn’s ability to promote adipogenesis.

## Discussion

**Figure 6:**
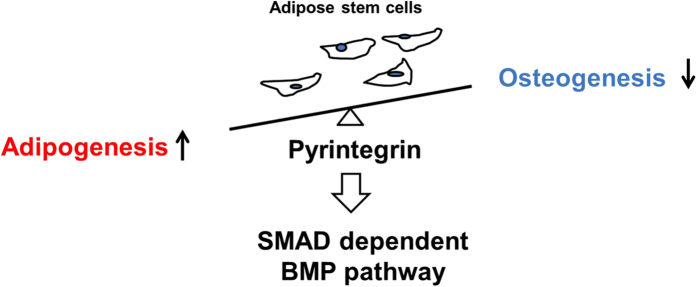
Proposed model for the role of Ptn signaling in hASCs. See discussion for details.

The present data provides the first experimental evidence that a small molecule, Ptn is potent in concurrently capable of promoting adipogenic differentiation and attenuating osteogenic capability of human ASCs by mediating SMAD dependent BMP signaling attenuation *in vitro* (Fig. 6). It further demonstrates that this small molecule, Ptn promotes adipose tissue formation from either transplanted human adipose stem/progenitor cells or host endogenous cells. Ptn’s ability to induce *in vivo* adipogenesis is supported not only by its promotion of crucial adipogenic genes including PPARγ and C/EBPα, but also by adipocytokines such as adiponectin and leptin. PPARγ, known as a key adipogenesis transcription factor, is activated by prostaglandins and anti-inflammatory agents including indomethacin and thiazolidinedione^46^,^47^,^48^. Ptn appears to signal differently than PPARγ agonists. By itself, Ptn fails to promote PPARγ gene expression and thereby does not directly promote adipogenesis. Nonetheless, Ptn robustly promotes adipogenesis in the presence of known adipogenic supplements including insulin, dexamethasone, and cAMP activators. Ptn’s ability to stimulate adipogenesis only in the presence of known adipogenic supplements is supported by the outcome of our two *in vivo* experiments in which adipogenesis is induced not only by Ptn primed adipose stem/progenitor cells, but also adipogenesis by host endogenous cells. Either way, adipogenesis within the microchannels of 3D printed scaffold suggests that transplanted cells or recruited host endogenous cells are the cell sources for adipose tissue formation. In this regard, Pyrintegrin acts similarly to IGF1^28^ that has been shown to orchestrate adipose tissue formation from endogenous cells in an existing adipogenic environment.

In multiple bone disorders, stem/progenitor cells differentiate towards adipogenic lineage at the expense of osteogenesis. PPARγ activation with TZDs promotes the differentiation of precursor cells into adipocytes and inhibits osteoblast differentiation^49^. By comparing Ptn with known PPARγ promoters such as Rosiglitazone by performing human PPARγ luciferase assay, we concluded that Ptn does not appear to be a classic PPARγ agonist. These data suggest that Pyrintegrin promotes adipocyte differentiation through a mechanism that is distinctively different from that of PPARγ agonists. The functional importance of BMP signaling in promoting adipogenesis is supported by Ptn’s ability to inhibit osteogenic differentiation and promote lipid accumulation *in vitro*. BMP responsive SMAD1/5/8 promotes osteogenesis by interacting with Runx2 to activate target gene transcription, leading to osterix induction^50^. Since the present data show that Ptn attenuates BMP-mediated SMAD1/5 activity and promotes adipogenesis even in osteogenic differentiation medium, we suggest that Ptn mediated BMP signaling might be adjunctive to Ptn-induced adipogenesis. The ability of Pyrintegrin to block SMAD1/5/8 phosphorylation in the absence and presence of BMP ligand suggests that Ptn attenuates BMP receptor kinase activity. Additional work will test Ptn effects in cells lacking BMP type II receptors and understand Pyrintegrin functions in the absence or presence of BMP receptors. Inhibition of BMP signaling is typically achieved by sequestering its ligands by administering soluble receptor extracellular domains or ligand-specific neutralizing antibodies^51^,^52^,^53^, or endogenous antagonists such as noggin or follistatin^54^,^55^,^56^. Hence, additional studies utilizing gain-of-function or loss-of function of BMP signaling in the presence of Ptn will likely elucidate BMP signaling in Ptn mediated adipogenesis.

Pyrintegrin induces lipid accumulation and adipogenic differentiation of adipose stem/progenitor cells in the presence of dexamethasone alone and in the absence of insulin and cAMP activators. Dexamethasone is a glucocorticoid and known as a potent stimulator for 3T3-L1 and TA-1 preadipocytes^57^,^58^,^59^,^60^. The glucocorticoid receptor is a member of the nuclear hormone receptor family and upon ligand binding can directly activate C/EBPδ transcription. The present findings demonstrate that the simultaneous activation of dexamethasone’s canonical signaling pathways, through the glucocorticoid receptor and Ptn through PPARγ and C/EBPα promotes adipogenic differentiation. In the absence of Dex alone treatment group, we wish to highlight the role of Dex based on the current literature. Dexamethasone (Dex) is known to alter bone marrow^61^,^62^ and muscle derived mesenchymal stem cells (MSCs) proliferation rate^61^, and enhances their osteogenic differentiation capability^61^,^63^. Dex alone does not induce adipogenic differentiation in MSCs over a 16-day culture period^64^ or in human adipose derived stem cells^65^. Dex and Rosiglitazone together are necessary and sufficient to induce adipogenic differentiation in MSCs^35^ and therefore we tested the effect of Pyrintegrin in the presence of Dex. Remarkably, lipid droplet formation and mineralization occur concurrently in response to Ptn treatment during osteogenesis.

Taken together, the present data show that Ptn promotes adipogenesis in an adipogenic environment either by Ptn-primed ASCs or host endogenous cells in Ptn-adsorbed scaffolds implanted in the inguinal fat pad. For translational studies, Ptn’s efficacy to promote endogenous adipogenesis without cell transplantation clearly offers advantages, similar to our previous work on tissue regeneration by cell homing, without the need for cell transplantation^43^,^44^,^66^,^67^. Pyrintegrin may offer potential advantages of tunable doses as a potential treatment for lipoatrophy, and an alternative to autologous fat transfer that has an intrinsic disadvantage of unpredictable post-operative volume reduction. Ptn attenuation of osteogenesis may have potential usage in promoting soft tissue healing and avoiding ectopic mineralization. Nevertheless, when using a small molecule the possibility of confounding effects from secondary targets should be considered, which we did not see in our *in vivo* studies due to the lowest effective concentration used. This study further warrants a more detailed and different dose analysis to maximize selectivity for effective adipogenic signaling.

## Methods

### Human adipose stem/progenitor cells

All experimental protocols were approved by Columbia University’s IRB approval and were carried out in accordance with their outlined guidelines and regulations. Human adipose stem/progenitor cells (hASCs) were isolated from lipectomy samples (anonymous female donors; age: 37 to 52 years old) per our prior methods^2^,^11^,^16^,^17^. We also obtained lipoaspirate-extracted ASCs from different donors from Dr. Farshid Guilak’s laboratory at Duke University Medical Center as a generous gift. An informed consent was obtained from all the human subjects whose tissue samples were utilized for this study design. Growth medium (DMEM) was defined as Dulbecco’s modified Eagle’s medium:Nutrient mixture F12 (DMEM/F12+Glutamax^TM^-I; Life Technologies, Grand Island, NY), 1% antibiotic (Antibiotic–Antimycotic 100X contains 10000 units of Penicillin, 10000 μg of Streptomycin and 25 μg of amphotericin B; Gibco, Invitrogen Corporation, Carlsbad, CA) and 10% Fetal Bovine Serum (FBS; Life Technologies, Grand Island, NY). Frozen cells were allowed to thaw in a 37 °C water-bath for 2 min and suspended in fresh DMEM. Cell suspension was centrifuged at 300 g for 5 min with cell pellet resuspended in fresh DMEM and plated in 5% CO_2_ at 37 °C. Medium was changed twice per week until the cells become confluent. Upon 80‐90% confluence, cells were washed, trypsinized, centrifuged, and resuspended in -DMEM as passage 1 (P1) and plated at a ratio of 1:4 or frozen in a freezing solution (80% (vol/vol) FBS, 10% (vol/vol) DMEM/Ham’s F-12 and 10% (vol/vol) Dimethyl sulfoxide (DMSO; Sigma-Aldrich, St. Louis, MO) and stored in liquid nitrogen, per our previous methods^2^,^11^,^17^. Cells were counted using 0.4% Trypan blue and Countess Automated cell counter (Invitrogen Corporation, Carlsbad, CA).

### Multi-lineage differentiation

Cells were treated with adipogenic induction medium (AIM) containing 1-μM dexamethasone (Dex), 10-μg/ml insulin, 0.5-μM isobutylmethylxanthine (IBMX) and 60-μM indomethacin (Sigma-Aldrich, St. Louis, MO) per our prior methods^2^,^10^,^11^,^16^,^17^. Human ASCs treated with growth medium and DMSO (vehicle) without adipogenic supplements represented DMEM cultures. Human ASCs treated with adipogenic induction medium represented AIM cultures. Human ASCs treated with adipogenic medium containing Pyrintegrin dissolved in DMSO represented AIM + Ptn cultures. For osteogenic differentiation, cells were treated with osteogenic induction medium (OIM) containing 100-nM Dex, 0.05-mM L-ascorbic acid-2-phosphate, and 10-mM β-glycerophosphate disodium salt hydrate (Sigma-Aldrich). All experiments were performed in triplicates.

### Pyrintegrin treatment of adipose stem/progenitor cells

To optimize the effective Ptn doses for adipogenic differentiation, Passage 5 (P5) ASCs were culture‐expanded in monolayer at a density of 5 × 10^3^ cells/cm^2^. At 80–90% confluence, ASCs were treated with DMEM, AIM and AIM supplemented with 2, 5 and 10 μM- Ptn. To understand the long-term effects of Ptn on adipogenic differentiation potential of hASCs, P5 hASCs were treated with DMEM, AIM, AIM supplemented with 2-μM Ptn (AIM + Ptn 2 μM) and DMEM supplemented with 2-μM Ptn (Ptn 2 μM only) for 28 days.

### Real Time qRT-PCR

Total RNA was isolated using PureLink RNA Mini kit (Life Technologies, Grand Island, NY) per manufacturer’s instructions. RNA samples were eluted in 30-μl DEPC‐water. Total RNA was quantified using NanoDrop 1000 Spectrophotometer (Invitrogen, Carlsbad, CA) and stored at −80 °C prior to reverse transcription. All RNA samples were reverse-transcribed using a High‐Capacity cDNA Reverse Transcription kit (Applied Biosystems, Foster City, CA). The cDNA was synthesized using 0.1–0.2 μg RNA to obtain 20-μL cDNA. All cDNA samples were stored at ‐20 °C until further use. For qPCR reactions, cDNA samples were mixed with TaqMan^®^ Universal PCR Master Mix (Applied Biosystems, Foster City, CA), primers, and nuclease‐free water per manufacturer’s protocol. PCR primers included peroxisome proliferator-activated receptor γ (PPARγ, Hs01115513_m1), and CCAT-enhanced binding protein α (C/EBPα, Hs00269972_s1) gene-specific FAM dye tagged primers. Osteogenic markers included Runt-related transcription factor 2 (RunX2, Hs00231692_m1), and Osterix (Sp7, Hs01866874_s1) gene-specific FAM dye tagged primers (Applied Biosystems, Carlsbad, CA). All mRNA was measured using ViiA™7 Real-Time PCR System (Applied Biosystems) in 25-μl reaction volume, with GAPDH as a house-keeping gene.

### Lipid staining

Human ASC cultures were washed with PBS (Sigma-Aldrich), fixed in warmed 4% paraformaldehyde solution (Fisher-Scientific, Pittsburgh, PA) for 10–30 min at room temperature. Cells were gently washed twice with PBS and then stained with LipidTOX^TM^ neutral lipid stain (Invitrogen) for 10–15 min per our prior methods.

### Adipogenesis assays

Human ASCs were plated at 5 × 10^3^/cm^2^ and differentiated in chemically defined medium for 28 days. Culture medium and lysed cell supernatant were individually collected on 14 and 28 days. Cells were lysed using 1% Triton-X solution (Sigma-Aldrich, St. Louis, MO). Cell culture lysate was collected by scrapping with a cell scraper (Fisher-Scientific) and briefly sonicated for 10 s using an ultrasonic water bath (Fisher-Scientific) on ice. Total secreted human adiponectin and leptin were measured using ELISA (R&D, Minneapolis, MN) per our prior methods^2^,^10^,^11^,^16^,^17^. Absorbance was read using a microplate reader (Biorad, Hercules, CA). DNA content was measured using Quant-iT™ PicoGreen^®^ dsDNA Assay Kit (Life Technologies) per our prior methods. A high-range standard curve of 1 μg/mL was used for measuring the DNA concentration of the samples per our prior methods. The samples were prepared in a 96 well black bottom plate (Fisher-Scientific) with absorbance read using microplate reader and standard fluorescein wavelengths (excitation ~480 nm, emission ~520 nm; Molecular Devices, Sunnyvale, CA). Total triglycerides and glycerol contents were measured by processing the sample lysate with Free Glycerol Determination kit (Sigma-Aldrich) per our prior methods^2^,^10^,^11^,^16^,^17^.

### Scaffold design and synthesis

Scaffold design and synthesis followed our previous methods to create 3D printed scaffolds with interconnecting microchannels^43^,^44^,^66^,^67^. Using 100 wt% poly-ε-caprolactone, PCL (Sigma-Aldrich Corp.,) with molecular weight ~65 KDa was 3D-printed using a bioplotter (EnvisionTec, Gladbeck, Germany). PCL pellets were first melt at 120 °C and then printed layer-by-layer as a cylinder (5 × 3 mm: diameter × height) using CAD software. Interlaid strands had diameters of 250–300 μm and interconnecting microchannels had a diameter of 400–500 μm. Scaffolds were sterilized in Ethylene Oxide (ETO) gas (Anprolene AN74i, Fisher, Pittsburgh, PA).

### Cell seeding in scaffolds

Cells were washed, trypsinized, counted and seeded at a density of 10 × 10^6^ cells/mL by mixing the cells with 3 mg/mL Collagen Type I solution (BD Bioscience, Sparks, MD). Collagen solution was allowed to crosslink for 1 h at 37 °C before *in vivo* implantation. Sample size per group was 6.

### *In vivo* implantation of cell-seeded scaffolds

All experimental protocols were approved by Columbia University’s Institutional Animal Care and Use Committee (IACUC) and were performed in accordance with their guidelines and regulations. Athymic nude male mice (7–8 wks old; Harlan, Indianapolis, IN) were anesthetized with intraperitoneal ketamine (100 mg/kg) and xylazine (5 mg/kg), and maintained at 1–3% isoflurane (Fisher-Scientific). The skin was disinfected with 70% Ethanol and wiped with Betadine and Povidone-Iodine (Fisher-Scientific). Following a linear midsaggital incision, subcutaneous pockets were created using fine scissors and housed all *in vivo* implanted scaffolds, followed by skin closure with a 4–0 P-3 vicryl coated undyed braided suture (Ethicon, Somerville, NJ). All mice received post-surgical Rimadyl IP injections for pain control (5 mg/kg, Pfizer, New York, NY). All scaffolds were retrieved after 4 wks following euthanasia with CO_2_ asphyxiation.

### *In vivo* implantation of Pyrintegrin adsorbed scaffolds

A total of 3 mg/mL neutralized collagen type I solution was prepared per our prior method^43^,^44^,^66^,^67^ with 10 μg/mL Ptn adsorbed. Ptn-adsorbed or Ptn-free collagen gel was infused into the scaffolds’ microchannels by pipetting, followed by collagen crosslinking for 1 h at 37 °C before *in vivo* implantation. Following Columbia University IACUC approval, C57BL/6 male mice (age: 10–11 wks, weight 15–25 g; Harlan, Indianapolis, IN) were fed with adjusted irradiated calories diet (Harlan). Given the mouse susceptibility to diet-induced obesity, the mice gained ~10–15 g in 6 wks (data not shown), after which we surgically implanted Ptn-adsorbed and collagen gel alone scaffolds in separate mice (N = 6 per group). On the day the surgery, the mice were anesthetized with 3–5% isoflurane. The skin was disinfected with 70% Ethanol, Betadine and Povidone-Iodine. An 1–1.5 cm long incision was made in the upper segment of the hindlimbs on both sides to expose the inguinal fat pad. A small pocket was created using fine dissecting scissors in this fat pad followed by implantation of Ptn-adsorbed or collagen gel only scaffolds, and closure of fat pad and skin using a 4–0 P-3 vicryl coated undyed braided suture. All mice received post-surgical Rimadyl IP injections for pain control (5 mg/kg, Pfizer). All scaffolds were retrieved after 4 wks following euthanasia with CO_2_ asphyxiation.

### Histology and immunohistochemistry

The retrieved scaffolds were embedded in optimal cutting temperature medium (Fisher-Scientific) in cryomolds and frozen in isopentane (Fisher-Scientific) pre-cooled in liquid nitrogen and processed for cryosectioning (Histoserv, Germantown, MD) into 8-μm sections. Frozen sections were fixated in 4% paraformaldehyde (Fisher-Scientific) at room temperature for 15 min, permeabilized with 0.25% Triton X-100 (Sigma-Aldrich, St. Louis, MO) and blocked with Licor Odyssey Blocking buffer (Licor) and stained with Oil Red O and hematoxylin.

### Human PPARγ reporter assay

PPAR Reporter assay kit (Indigo Biosciences, State College, PA) utilizing non-human mammalian cells engineered to express constitutive, high-level expression of human PPARγ, a ligand-dependent transcription factor. These reporter cells include the luciferase reporter gene functionally linked to a PPARγ-responsive promoter. Thus, quantifying changes in luciferase expression in the treated reporter cells provides a sensitive surrogate measure of the changes in PPARγ activity. Rosiglitazone was used as a positive control due to its ability to bind to the nuclear PPARγ receptor. As per manufacturer’s instructions, the reporter cells were plated and immediately treated with Rosiglitazone or different Ptn doses. No adipogenic inductive factors were used in this assay. Following overnight incubation at 37 °C, the treated medium was discarded followed by addition of 100-μl luciferase detection reagent (LDR), and incubation at room temperature for 15 min. Light intensity emitted was quantified using a plate-reading luminometer.

### Western blot

Cells were treated with growth (DMEM), adipogenesis induction medium (AIM), AIM containing 2-μM Ptn (AIM + Ptn) or 2-μM Ptn supplemented DMEM for 1 h, and then harvested in lysis buffer (50-mM Tris-HCl; pH 8), 1% NP40, 150-mM NaCl, phosphatase Inhibitor, protease Inhibitor for 20 min on ice. Cell extracts were centrifuged at 12,000 × g for 20 min at 4 °C. Protein concentration was measured using a Bradford Protein assay (Bio-Rad, Hercules, CA). A total of 20-μg protein was denatured at 70 °C for 10 min and separated on a 4–12% NuPAGE Novex Bis-Tris gel electrohoresis (Invitrogen), followed by transferring of protein onto a PVDF membrane (Biorad). The membrane was blocked using Odyssey blocking buffer, rocked on a shaker for 30 min, and immunoblotted overnight with phospho-p42/p44 MAPK, phospho-SMAD1/5, SMAD1 primary antibodies at 4 °C (Cell Signaling Technologies, Danvers, MA) and β-Actin (Santa Cruz Biotechnology, Paso Robles, CA). Specifically bound primary antibodies were detected by incubating for 1 h with ALP conjugated secondary antibodies and enhanced using 1-Step NBT/BC1P (Fisher Scientific).

### Immunobloting of BMP and TGFβ-responsive SMAD phosphorylation

ASCs were plated at 5 × 10^3^ cm^2^ per 10-cm petridish for SMAD signaling and MAPK p38 signaling. For SMAD1/5 and MAPK p38 signaling studies, the cells were serum-starved overnight and treated with DMEM/F12 medium and vehicle or DMEM/F12 medium and vehicle containing 30 ng/mL BMP4 and varying Ptn doses (0, 0.2, 0.5, 1, 2, 5, and 10 μM) for 1 h. For SMAD2 signaling studies, the cells were serum-starved overnight and treated with DMEM/F12 medium and vehicle or DMEM/F12 medium and vehicle containing 0.5 ng/mL TGFβ1 and varying Ptn doses for 1 h. Cell extracts were mechanically homogenized in lysis buffer, separated by NuPAGE Bis-Tris gels, immunoblotted with anti-phospho-SMAD1/5, anti-SMAD1, anti-phospho-SMAD2, anti-phospho-p38 MAPK, anti-p38 MAPK (Cell Signaling) or anti-β-Actin primary antibodies, and Alexa Flour 680 goat anti-rabbit IgG or Alexa Flour 680 goat anti-mouse IgG (Invitrogen) and visualized using Odyssey Infrared Imager (Licor).

### Alizarin Red staining

ASCs were seeded onto a 24 well plates and treated with osteogenesis induction medium (OIM) with or without 2-μM Ptn for 3 wks. Cells were fixed in warmed 4% paraformaldehyde and stained with Alizarin Red S stain (Sigma-Aldrich) for 5 min as per manufacturer’s protocol.

### Dexamethasone treatment

ASCs were seeded at 5 × 10^3^ cells/cm^2^ in 24 well plates. After cells were exposed to 2-μM Ptn in DMEM with different dexamethasone doses (0.1 to 10 μM) for 3 wks, culture medium was removed with cells stained for lipid visualization.

### Statistical analysis

Upon confirmation of normal distribution, all quantitative data were analyzed using a one‐way analysis of variance (ANOVA) with post‐hoc Bonferroni tests to determine significant differences between or within groups, using a statistical package (SPSS, Chicago, IL). Results were considered significant for p < 0.05.

## Additional Information

**How to cite this article**: Shah, B.S. *et al*. Pyrintegrin Induces Soft Tissue Formation by Transplanted or Endogenous Cells. *Sci. Rep.* **7**, 36402; doi: 10.1038/srep36402 (2017).

**Publisher's note:** Springer Nature remains neutral with regard to jurisdictional claims in published maps and institutional affiliations.
